# Psychometric properties of the Russian version of the Pediatric Daytime Sleepiness Scale (PDSS)

**DOI:** 10.1016/j.heliyon.2019.e02134

**Published:** 2019-07-25

**Authors:** C. Randler, S.N. Kolomeichuk, A.V. Morozov, D.A. Petrashova, V.V. Pozharskaya, A.A. Martynova, L.S. Korostovtseva, M.V. Bochkarev, Y.V. Sviryaev, M.G. Polouektov, C. Drake

**Affiliations:** a University of Tuebingen, Department of Biology, Auf der Morgenstelle 24, D-72076 Tübingen, Germany; b Laboratory of Genetics, Institute of Biology of the Karelian Science Center of the Russian Academy of Sciences, Petrozavodsk, Russia; c Laboratory of Ecological Physiology of Animals, Institute of Biology of the Karelian Science Center of the Russian Academy of Sciences, Petrozavodsk, Russia; d Kola Science Center of the Russian Academy of Sciences, Apatity, Murmansk Region, Russia; e Almazov National Medical Research Center, Saint-Petersburg, Russia; f I.M. Sechenov First Medical University, Moscow, Russia; g Henry Ford Hospital Sleep Disorders and Research Center, Detroit, MI, USA

**Keywords:** Psychiatry, Clinical psychology, Physiology, Pediatrics, Psychometrics, Sleep duration, Pediatric daytime sleepiness scale, Sleepiness, Children

## Abstract

Insufficient sleep could severely impair both cognitive and learning skills. More prominent changes are found in children and adolescents. Tools used to estimate sleepiness in the adult population are commonly inappropriate for children. The objective of our study was to provide a reliable instrument to measure excessive sleepiness for upcoming studies in Russian-speaking children, applying the Russian version of Pediatric Daytime Sleepiness Scale (PDSS). The following tasks were resolved in our study: translation, validation, and analysis of psychometric properties of the Russian adaptation of the PDSS by standard tests. After the semantic validation of the instrument through a multi-stage translation process we checked its psychometric validation. A total of 552 students, consisting of N = 285 for the exploratory factor analysis (EFA), N = 267 for the confirmatory factor analysis (CFA) and N = 204 for test-retest analysis of public elementary schools located in Northern Russia completed the PDSS and Munich Chronotype Questionnaire to estimate sleep parameters in the classroom during the lessons. Response rate was 90%; excluded cases contained no data. Further, 204 of our participants completed the PDSS in a 3 months interval to check the test-retest reliability. Internal consistency was measured by Cronbach's alpha coefficients and CFA was used to test factorial validity of the tool. Concurrent validity and test-retest reliability were assessed via intra-class coefficient. Internal consistency of the PDSS scale was high (Cronbach's α = 0.8). The construct validity of the PDSS was supported by CFA (factor loadings were from 0.438 to 0.727) and the test-retest reliability demonstrated by the intra-class coefficient was 0.70. The total PDSS score was independent of sex. The mean total value of PDSS was 11.95 ± 6.24. Higher scores on PDSS were negatively correlated with sleep duration. Thus, the construct validity of the instrument remains valid and could be used for Russian-speaking youth samples in the evaluation of daytime sleepiness. It could be useful in future applications by sleep scientists and health practitioners.

## 1 Introduction

Latest recommendations of the National Sleep Foundation propose that children need at least 7 h of sleep per night (Hirshkowitz et al., 2015). However, modern children obtain less sleep than required (Fallone et al., 2002). Daytime sleepiness is excessive among children and adolescents, which is of great concern for health and education professionals in recent years (Dorofaeff and Denny, 2006). One reason for the increased concern is the recognition that this group tends to have insufficient amounts of sleep in general (Carskadon et al., 1981; Carskadon, 1990). The limited amount of sleep was found to be associated with increased level of sleepiness during the day, with many behavioral and emotional disturbances and academic impairment (Gibson et al., 2006). In the clinical practice, many sleep disorders characterized by sleepiness are more common than previously assumed. It is important to assess sleepiness and sleep disorders in children and adolescents. Wolfson and Carskadon reported a relationship between adolescent sleep and their performance (Wolfson and Carskadon, 1998, 2003). Eliasson found no clear association between sleep quantity and academic achievement (Eliasson et al., 2002).

Several works have shown that light pollution as well as social interactions and other behavioral patterns form children's' lifestyles induce a shift to nighttime activity, while school schedules demand being fully awakened early in the morning (Vollmer et al., 2012; Porcheret et al., 2018). This discrepancy leads to reduced weekday sleep time and an accumulating sleep debt (Carskadon et al., 1981; Kolomeichuk et al., 2016; Randler et al., 2019).

As sleep loss and sleep disorders have severe consequences for adolescents, there is an urgent need for easily available and sensitive measures of sleepiness for use in this age group. This could help with the identification of at-risk individuals and to track treatment progress over time. Such subjective measures of sleepiness complement objective measures (like actigraphy) especially when time consuming assessment is not feasible.

To assess sleepiness in both research and clinical settings standardized procedures, such as the multiple sleep latency test (MSLT) and the maintenance of wakefulness test (MWT), have been developed. These applications are based on equipment, much time and labor. On the other hand, various self-rating scales were developed for the evaluation of sleepiness. The most widely used instrument for this purpose is the Epworth Sleepiness Scale (ESS) for adult sleepiness (Johns, 1991). The ESS has been validated in different clinical populations (Guilleminault et al., 1988; Hagell and Broman, 2007) and in different languages (Bloch et al., 1999; Cho et al., 2011; Izci et al., 2008). It measures the level of sleepiness by listing eight daily life situations for which raters judge the likelihood of them falling asleep (Guilleminault et al., 1988; Hagell and Broman, 2007). Meanwhile, modified version of ESS has been used in children and adolescents to examine the relationship of sleep-disordered breathing and hyperactivity (Melendres et al., 2004) and psychological functioning and sleep (Moore et al., 2009; Imani et al., 2018). Noteworthy, only Imany with colleagues reported their sample size. Moore et al. (2009) used the terms sleep duration, variation in sleep duration, and sleepiness as a proxy for sleep disorders. To develop sleepiness rating scales for children and adolescents, Drake et al. introduced a validated measure for sleepiness in children, PDSS (Drake et al., 2003). Using this scale, daytime sleepiness was found to be related to lower academic performance and other negative school-related outcomes (Perez-Chada et al., 2007). Among the few self-rating scales available, the PDSS revealed a good reliability and a high validity in a sample of middle-school children with an age range from 11 to 15 years. The PDSS contains eight questions concerning sleep-related behaviors for which the frequency of these behaviors is rated on a 5-point Likert scale. It is a self-assessment that verifies some situations related to sleeping habits and problems (Pereira et al., 2010), as well as the attention of adolescents (Perez-Lloret et al., 2013). The PDSS is frequently used in different studies (Drake et al., 2003; Huamaní and Castro, 2014; Inocente et al., 2014; Jarrin et al., 2014; Langberg et al., 2013; Gu et al., 2015; Maganti et al., 2006; Perez-Chada et al., 2007). The PDSS was translated into German (Schneider and Randler, 2009), Brazilian Portuguese (Felden et al., 2016), Turkish (Bektas et al., 2016), Chinese (Yang et al., 2010), Korean (Rhie et al., 2011) and into Japanese (Komada et al., 2016).

Our study was carried out to estimate excessive sleepiness and the PDSS values in both urban and rural areas in Northern European part of Russia predominantly between 7 and 12 years old during our framework project “Sleep Health in Russian Arctic”. No previous works have tested Russian version of this scale. To complete the study goal, the PDSS was distributed to a larger children cohort to establish the reliability of the tool.

## 2 Materials and methods

### 2.1 Translation procedure

Written permission was granted by e-mail from author (Dr. Christopher Drake) to adapt the PDSS into Russian and to use it in further studies. The translation process was split in several steps. First, the English version was translated into the Russian language by two bilingual native translators (forward translation). Second, both versions were combined into the merged Russian version by the two translators under supervision of the research project coordinator. Next, the merged Russian version was then translated back into English by two native English professional translators. They worked separately and independently from each other and were not informed about the original English version of the PDSS. Further, all translated variants in both forward and backward translations were represented and discussed by an expert review board (pediatrician, psychologist, psychiatrist, psychometrician, and certified nurse) dealing with their linguistic, experiential and conceptual equivalencies. All corrections were done to clarify the tool for a better interpretation. The provisional version was preliminary tested on the randomly selected group of children (n = 30; 14 males). After completion the questionnaire, these children were interviewed to evaluate what they thought about each item, its scoring as well as a guideline. The final version of the PDSS-RUS was applied to 552 children to evaluate psychometric properties of the instrument. Then, the final version represented the general agreement of the whole research team involved in this procedure. After the linguistic validation of the PDSS-RUS with a multi-step translation methodology mentioned above, we conducted a psychometric validation.

### 2.2 Sample size calculation

CFA analysis was the most complicated amongst the analyses in our study. Thus, we used it to evaluate and justify our sample size. According to the literature, a sample size equal to or greater than 200 is considered as the stable estimate (Kline, 1998; Fang et al., 2015). Also, the rule of thumb claims the ratio of *n* per variable for the CFA is 20 (Myers et al., 2011). As for the PDSS, the estimated preferred sample size is 160 based on the rule of thumb. As a result, we believed that a sample size of more than 200 would be enough.

### 2.3 Participants and procedure

The study was performed from March to October of 2018. Schools from six different settlements located in North-Western Russia participated in the study during our framework project “Sleep Health in Russian Arctic”. The Republic of Karelia consists of 18 educational districts and Murmansk region consists of 6 educational districts. After that 3 elementary schools were randomly selected. Then, one class per grade was randomly selected from each of the determined schools. Since there are three grades per school, a total of 216 classes were included. This represented a sample of children from both urban and rural areas in Northern European part of Russia predominantly from 7 to12 years old. Inclusion criteria for participants were: ability to read and comprehend questionnaires in Russian and written, informed consent with the study protocol. Further, children and their parents were informed about the aims of the study, and that all data would be gathered anonymously. Participants' legal guardians signed the written informed consent. Children completed self-rating questionnaires individually in their classroom during an official school lesson, covering sleep-related information. To warrant data security, all participants put their completed questionnaires directly to the researcher; thus, neither classmates nor teachers were able to know participants’ answers. The Medical Ethics Committee (Karelian Health Development and Petrozavodsk State University) approved the study according to Protocol # 41 from 06.03.2018, which was completed according to the ethical principles described in the Declaration of Helsinki and its later amendments. Approved final version of the PDSS (see Appendix below) was distributed to all participants in each school. The questionnaire was presented to the students in a classroom after the written consent had been obtained from the parents and the school authorities. The students were voluntary to answer the questionnaire. Participants and their representatives were notified about the purpose and the nature of the study in written form. The study was conducted anonymously, and the participants were granted confidentiality of the data. The use of data for scientific purposes only was guaranteed.

The total sample size was 552 students, consisting of N = 285 for the EFA, N = 267 for the CFA and N = 204 for test-retest reliability.

### 2.4 Study I

The focus sample comprised 400 students. Of these, 60 (10%) were absent from school on the day of the survey and 337 students agreed to participate in the study. In 52 cases (15. 2%) information on key variables was missing and they were eliminated from analysis. The final study sample included 285 subjects. Mean age in the sample was 11.20 ± 2.75 years. Girls dominated in our sample (129 boys, 156 girls).

### 2.5 Study II

Study II consisted out N = 267 students (129 boys, 138 girls), mean age 10.37 ± 1.97 years (85% between 7-12 years). This data set was used for the CFA.

### 2.6 Study III

To examine test-retest reliability, 204 randomly selected participants (86 boys, 118 girls) were randomly chosen to complete the PDSS-RUS 3 weeks later once again (the second measurement took place during the same semester). Mean age was 9.75 ± 1.46 years.

### 2.7 Instrument

The PDSS represents an 8-item questionnaire elaborated to assess extreme daytime sleepiness in schoolchildren which was translated into Russian, and then tested for comprehension (See both Appendices A & B). The interviewed person rates the frequency of these behaviors on a 5-point Likert scale from 0 (never) to 4 (always). Rankings on all the items were calculated to get the total score from 0 to 32. The higher score means greater daytime sleepiness. Moreover, daytime sleep episodes were retrieved from self-assessed children sleep dairies.

To retrieve the information on sleep length each respondent filled in the Munich Chronotype Test Questionnaire. Translated Russian version was approved by the author of the original tool and successfully applied before to schoolchildren (Borisenkov et al., 2010; Kolomeichuk et al., 2015). This instrument calculates several sleep parameters. However, we used only data about sleep duration in our analyses. Completed surveys were processed as previously described (Roenneberg et al., 2004). Sleep duration was calculated as the difference between time of wake up and time of asleep [Sleep duration = time of wake up – time of asleep] in hours. Daytime sleepers (nappers) were asked whether they nap and if, how long these naps are.

### 2.8 Statistical analyses

Construct validity was examined through EFA and CFA. The following adjustment indicators were used as criteria: Comparative Fit Index (CFI) and Tucker Lewis Index (TLI) with values ≥ 0.90, Root Mean Square Error of Approximation (RMSEA) and Standardized Root Mean Square Residual (SRMR) with values of 0–0.8 (Brown, 2014).

Item 3 is inverse coded and was recoded for reliability analysis and sum scores. Cronbach's α was used for correlation between items of the PDSS and the full scale. An EFA with principal component analysis was applied to analyze the factor structure. Statistical analyses were performed using SPSS 25.0 (for reliability, EFA, correlation analysis and T-Tests). AMOS 25.0 was used for the CFA. We used the parallel analysis engine to calculate random factors for a parallel analysis based on Raiche (2010). In this tool, number of cases and items are input and random eigenvalues are constructed. We used principal components analysis and 1000 iterations. The sample size was that of sample 1. For details of the program, see (https://analytics.gonzaga.edu/parallelengine/_w_5170816bafa71f6e43d8f944c0600831a38ae604100df881/). For the intra-class coefficient, ICC, we used a two-way mixed model with absolute agreement, based on the average measure. The choice of the respective model was based on the suggestions of Koo & Li (2016).

## 3 Results

### 3.1 Translation and cultural adaptation

Appendix A represents both translation as well as back-translation process undertaken of the PDSS questions.

The questions reflected clarity in the comparison between translation and back translation; the proposed final version of the questions was contextually similar to the original one regarding the language expressions. However, an adaptation was carried out in questions #3, 4, 7 and 8 for better understanding, based on the advice of the area specialist and the focus group of children. The review board experts were asked about the clarity and linguistic appropriateness considering the understanding of children and adolescents in the age range of the original tool. Based on this assessment, the word “*ϑнимательный*” (aware) was added to the word “*бодрый*” (alert) in question three, due to a better understanding by children and adolescents. In question # 2 experts advised to pay attention to the process. Question # 4 word “*без настроения*” (grumpy) was expanded for more precise tired, irritable and in a bad mood “*устаϑ♯ий и раздражительный*”. In addition, in this assessment, somnologists suggested adapting question #7: “How often do you need someone to wake you up in the morning?” The expert suggested considering other options of awakening, such as an alarm devise. The suggestion was considered valid and adapted in the final version of the scale. Somnologists proposed to confine question # 8 How often do think you need more sleep as sleep shortage for better understanding the item.

Furthermore, in the final version of the scale, question two was adjusted considering the terms “sleepy” and “drowsy.” These terms were considered synonyms in the Russian language, and it is believed that the redundancy used in the English language is not necessary for the appropriate understanding of the question in Russian. Thus, the final question was “How often do you feel sleepy while doing your homework?”. The scale is presented in Appendix B.

### 3.2 Study I – EFA

PDSS scores in the sample ranged from 0 to 29. Mean values in our study were 11.95 ± 6.24 (See Table 1). The PDSS was found to have a good internal consistency, with Cronbach alpha of 0.802 of the PDSS scale in the full sample. Mean item-scale correlation was 0.511 and ranged from 0.322 (item 3) to 0.600 (item 8). Mean inter-item correlations were 0.334 and ranged from 0.175 to 0.470. Cronbach's alpha for the original publication were 0.8.

**Table 1:** Sample characteristics.

Variables	Entire sample (n = 552)	EFA sample (n = 285)	CFA sample (n = 267)	Test-retest sample (n = 204)
Age (Year); Mean ± SD	10.79 ± 2.36	11.2 ± 2.75	10.37 ± 1.97	9.75 ± 1.46
Sex (Male); n (%)	258(46,8)	129(45.3)	129(48.3)	86(42.2)
PDSS score; Mean ± SD	12.71 ± 6.03	11.95 ± 6.24	13.47 ± 5.79	12.75 ± 6.06
Sleep duration, h; Mean ± SD	8.46 ± 1.6	8.13 ± 1.47	8.79 ± 1.72	8.94 ± 1.19
Daytime nappers, n (%)	12.4	8.4	16.3	12
Daytime sleep duration, h; Mean ± SD	0.33 ± 0.64	0.39 ± 0.61	0.26 ± 0.68	0.21 ± 0.63

PDSS = Pediatric Daytime Sleepiness Scale; EFA – exploratory factor analysis; CFA - confirmatory factor analysis.

Concerning the exploratory factor analysis, there was only one eigen-value greater than one, suggesting that all items load onto a single factor. This corroborates the findings of the reliability analysis. All factor loadings were higher than 0.4 (Table 2). Based on a parallel analysis, the three highest random Eigenvalues were 1.25, 1.16, and 1.087. As the second Eigenvalue of the present analysis was 0.901, this clearly suggest having only one factor to be extracted. We found that PDSS scores correlated with age and daytime sleep episodes. An inverse correlation was observed for sleep duration during the last night with PDSS scores (Table 3). This suggests that adolescents are sleepier during the day when their nighttime sleep duration is shorter. There were also significant correlations between age and PDSS scores with higher scores being correlated with older age, so that older students had higher daytime sleepiness (Table 3). The duration of daytime napping was marginally non-significant and weakly associated with PDSS score. Daytime sleepers have a higher PDSS scores, and shorter nighttime sleep is related to daytime napping. According to our data no significant gender effect was detected (T = -0.802, df = 283, P = 0.432).

**Table 2:** Exploratory factor analysis item loadings.

Item #	Item description	Factor loading
1	How often do you think you need more sleep?	0.727
2	Drowsy/asleep during homework	0.704
3	Trouble getting out of bed in the morning	0.702
4	Fall asleep/drowsy during class	0.690
5	Tired and grumpy during the day	0.662
6	Need someone to awaken you	0.624
7	Fall back to sleep after being awakened	0.609
8	Usually alert during the day (reverse coded)	0.438

**Table 3:** Correlations between PDSS scores and sleep-wake variables and age.

		PDSS score
Age	Pearson, r	0.209
	p (2-tail)	<0.001
Sleep duration last night	Pearson, r	-0.321
	p (2-tail)	<0.001
Daytime sleep	Pearson, r	0.212
	p (2-tail)	<0.001
Duration daytime sleep	Pearson, r	0.110
	p (2-tail)	0.064

### 3.3 Study II – CFA

**Fig. 1:**
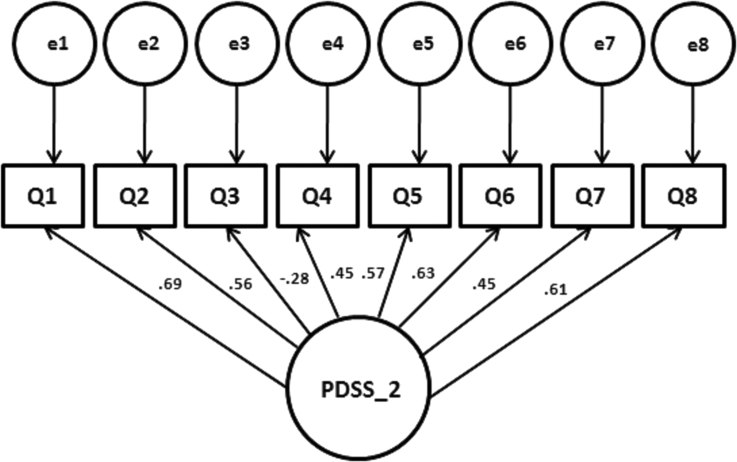
Graphical representation of the confirmatory factor analysis of the PDSS in Russian children. Notes: The factor was represented by the circle (PDSS). The observed variables (indicators/questions) were represented by rectangles. The factor arrows up to the observed variables represent the factorial loads.

Cronbach's α of the second sample was 0.76. Since there was no more than one factor to be extracted, a CFA was performed on a second sample. We calculated a comparative factor analysis with AMOS to the posited one-factor-structure of the PDSS. The total model received significance (Chi-square = 40.193, df = 20, p = 0.005). The loadings are depicted in Fig. 1. The model shows a good to acceptable fit. CMIN/DF was 2.010 [should be close to 2], RMSEA was 0.062 [0.00–0.05] (90%CI from 0.033 to 0.089 [close to 0], PCLOSE 0.222 [0.1–1.0], RMR 0.078 [0.>0.05], GFI 0.961[0.95–1.0], AGFI 0.930 [0.9–1.0], TLI 0.922[0.9–1.0], CFI 0.944 [0.95–1.0]. These data generally suggest a good fit of the model structure, again highlighting that the Russian version of the PDSS can be considered as uni-dimensional.

### 3.4 Study III – test-retest reliability

Test retest reliability was found to be high with a correlation of 0.708 (p < 0.001, N = 204); Cronbach's α of the first sample was 0.797, and 0.755 for the second sample. The PDSS score was slightly higher in the second sample and changed from 11.90 (±6.34) to 12.75 (±6.06; T = -2.552, p = 0.011). Intra-class coefficient (averaged) was 0.82 (95% CI from 0.768 to 0.868).

## 4 Discussion

The present study has two aims: adaptation of the PDSS into a Russian version and analysis of the factor structure of it. The best to our knowledge, this the first study describing psychometric properties of the PDSS when applied in Russian children. It should be noted that the Russian version of the PDSS exhibited good validity in young schoolchildren aged 7 to 12. Moreover, the scale had good internal consistency, with Cronbach alphas agreeing with values for the original publication (Drake et al., 2003). The reliability of the tool is acceptable and in good agreement with previous studies done with the PDSS in different countries (Drake et al., 2003; Komada et al., 2016; Maganti et al., 2006; Esposito et al., 2013; Rhie et al., 2011). Also, mean inter-item correlations demonstrated that the tool measures a broader and not too small a construct, which is supported by the one-factor solution of the exploratory factor analysis. The model was constructed with only one factor, as proposed by initial version (Drake et al., 2003) and also verified in Turkish and Brazilian Portuguese versions that both used the CFA for validation of the construct (Bektas et al., 2016; Ferrari Junior et al., 2018). Our model is good compared to the values for a good fit from Schermelleh-Engel et al. (Schermelleh-Engel et al., 2003).

More than 1000 articles have been found dealing with children and adolescent sleepiness dated from 2000 to 2018. Most of the studies used the PDSS as an instrument to assess sleepiness related to sleep pathologies (respiratory, neurological, and developmental disorders) and to assess sleepiness side effect monitoring in pharmacological treatments. In several studies, the authors applied the scale to healthy children and adolescents (Drake et al., 2003; Perez-Chada et al., 2007; Felden et al., 2016). Scale scores ranged from 6.7 to 25.7 showing a tendency for excessive daytime sleepiness in some works. Also, higher scores on the PDSS were associated with reduced total sleep time, poorer school achievement, weaker anger control, and frequent illness in different countries (Rhie et al., 2011; Perez-Chada et al., 2007; Yang et al., 2010). Our PDSS scores were in good agreement with data reported previously for healthy children from US (Maganti et al., 2006), Argentina (Perez-Chada et al., 2007), China (Gu et al., 2015) and Italy (Esposito et al., 2013). It should be noted that mean age in the sample was 11.20 years. That explains why PDSS scores received in our study are lower than those reported for Brazilian (Ferrari Junior et al., 2018) or Argentinean children (Perez-Chada et al., 2007). Our sample is younger than the Brazilian and Argentinean ones. Girls slightly dominated in our sample that corresponds to the demographic structure in the Russian population.

We found that PDSS scores were higher in older subjects. That is compatible with previous findings by different groups (Komada et al., 2016; Perez-Chada et al., 2007). Our data revealed that age is associated with daytime sleepiness, therefore mirroring the level of pubertal development. Thus, older adolescents have to sleep more than younger ones to reach the same level of alertness and cognitive performance (Randazzo et al., 1998; Gibson et al., 2006; Carskadon et al., 2004; Stickgold, 2005). However, in a recent study Feinberg and Campbell proposed that increased daytime sleepiness of adolescents is a consequence of adolescent brain reorganization decreasing the intensity of waking brain activity. Surprisingly, these brain changes are unrelated to pubertal maturation but are strongly associated with age (Feinberg and Campbell, 2010).

The PDSS scores in the retest sample were slightly higher, which could be related to the fact that the season was progressing and the daylight changes in these high latitudes. It affects sleep duration as well as daytime excessive sleepiness. Also, it shows that sleepiness is not a perfectly stable trait and may also have some fluctuations, as, e.g., it was reported by Schneider and Randler concerning the transition into daylight saving time (Schneider and Randler, 2009).

An inverse correlation was observed for sleep duration last night with PDSS scores. This suggests that adolescents are compensating sleep debt during the next day by having a nap.

Future research envisions a comparison for weekend and weekday sleep schedules with the Russian version of PDSS. Moreover, sleep hygiene behaviors are important in sleeping (Lin et al., 2018; Strong et al., 2018). Given the importance of the sleep hygiene for children, it will be our next step in the project.

Both parent and teacher reports are widely used in studying children (Kahn et al., 1989; Jonns, 1991), but it could be better when children themselves report their sleep problems to avoid parental monitoring during the administration of the questionnaire and allow direct responses from teenagers. Therefore, the PDSS is a useful tool in measuring sleepiness in school environments.

Russia is the largest country in the world consisting of 9 time zones. We conducted our study in the same time zone during short time range to avoid bias related to school schedule as well as day length, but it would be a fascinating study extending PDSS studies across the different time zones in Russia. We conclude that Russian version of PDSS remains valid and confirms PDSS factor structure with only one factor and also is applicable in the Russian version with a sample of adolescents. Psychologists, nurses and other professionals can use the Russian version of PDSS for research and evaluation of daytime sleepiness and its influencing factors.

### 4.1 Limitations

Our study has several limitations. First, the small number of participants, but still sufficient for a psychometric analysis, especially a factor analysis. Future studies should include additional measures of circadian preference as well as objectively measured data about the sleep-wake cycle.

## Declarations

### Author contribution statement

Christoph Randler: Conceived and designed the experiments; Analyzed and interpreted the data; Contributed reagents, materials, analysis tools or data; Wrote the paper.

Sergey Kolomeichuk: Conceived and designed the experiments; Performed the experiments; Analyzed and interpreted the data; Contributed reagents, materials, analysis tools or data; Wrote the paper.

Artem Morozov (AM): Performed the experiments; Analyzed and interpreted the data; Contributed reagents, materials, analysis tools or data; Wrote the paper.

Dina Petrashova, Viktoria Pozharskaya: Performed the experiments; Analyzed and interpreted the data; Contributed reagents, materials, analysis tools or data.

Alla Martynova: Performed the experiments; Analyzed and interpreted the data; Contributed reagents, materials, analysis tools or data; Wrote the paper.

L. S. Korostovtseva, M. V. Bochkarev: Contributed reagents, materials, analysis tools or data; Wrote the paper.

Y. V. Sviryaev, Mikhail Poluektov, Christopher Drake: Performed the experiments; Analyzed and interpreted the data.

### Funding statement

We acknowledge support by Deutsche Forschungsgemeinschaft and Open Access Publishing Fund of University of Tübingen. The study carried out under state order (project No 0218-2019-0077) and (No 0218-2019-0073). Also, research got partial support from PORA (Project Office for Arctic Development, Moscow, Russia) non-profit Institution.

### Competing interest statement

The authors declare no conflict of interest.

### Additional information

No additional information is available for this paper.
