# Supplementary material for: Psychometric properties of the Russian version of the Pediatric Daytime Sleepiness Scale (PDSS)
Source: Heliyon. 2019 Jul 25;5(7):e02134. doi: 10.1016/j.heliyon.2019.e02134 (PMC6661285; doi:10.1016/j.heliyon.2019.e02134)
Supplement: Appendix_A [file mmc1.pdf]

APPENDIX A

Cultural adaptation of the Russian version of the Pediatric Daytime Sleepiness Scale (PDSS)

Original English version	Translation	Back-translated version	Final Russian version
1.How often do fall asleep or get drowsy during class periods?	Как часто вы засыпаете или чувствуете сонливость во время занятий?	How often do you fall asleep or feel sleepy in class?	Как часто вы засыпаете или чувствуете сонливость во время занятий?
2. How often do fall asleep or get drowsy during your homework?	Как часто вы засыпаете или чувствуете сонливость, выполняя домашнее задание?	How often do you fall asleep or feel sleepy when doing homework?	Как часто вы засыпаете или чувствуете сонливость во время выполнения** домашнего задания?
3*. Are you usually alert most of the day?	Чувствуете ли Вы себя обычно бодрым**?**	Do you usually feel alert for most of the day?	Бодры ли вы обычно большую часть дня**?
4. How often are you ever tired or grumpy during your day?	Как часто Вы чувствуете себя уставшим и без настроения в течение дня?	How often do you feel tired, irritable, and in a bad mood during the day?	Как часто Вы чувствуете себя уставшим и раздражительным** в течение дня?
5. How often do you have trouble getting	Как часто Вы испытываете	How often do you find	Как часто Вы испытываете

out of bed in the morning?	проблемы при пробуждении утром?	it difficult to wake up in the morning?	трудности пробуждения по утрам**?
6. How often do you fell back to sleep after being awaken in the morning?	Как часто Вы снова засыпаете после пробуждения утром?	How often do you fall back to sleep after waking up?	Как часто Вы снова засыпаете после того как проснетесь** утром?
7. How often do you need someone to awaken you in the morning?	Как часто Вам нужна посторонняя помощь чтобы проснуться утром?	How often do you need help from others to wake up in the morning?	Как часто Вам нужна посторонняя помощь чтобы проснуться утром?
8. How often do think you need more sleep?	Как часто вы думаете что Вам нужно больше спать?	How often do you think that you need more sleep?	Как часто вы думаете что Вам не хватает сна**?

***This item has reverse score**

****Stressed words are changed in final version**
