# Supplementary material for: Psychometric properties of the Russian version of the Pediatric Daytime Sleepiness Scale (PDSS)
Source: Heliyon. 2019 Jul 25;5(7):e02134. doi: 10.1016/j.heliyon.2019.e02134 (PMC6661285; doi:10.1016/j.heliyon.2019.e02134)
Supplement: Appendix_B [file mmc2.doc]

APPENDIX B

**Детская Шкала Дневной Сонливости (PDSS)**

**Баллы: 4 = Всегда** **3 = Часто** **2 = Иногда** **1 = Редко** **0 – Никогда**

Ответьте на следующие вопросы как можно точнее, выбрав только один ответ:

1. **Как часто вы засыпаете или чувствуете сонливость во время занятий?**

Всегда Часто Иногда Редко Никогда

1. **Как часто вы засыпаете или чувствуете сонливость, выполняя домашнее задание?**

Всегда Часто Иногда Редко Никогда

**3.* Чувствуете ли Вы себя обычно бодрым большую часть дня?**

Всегда Часто Иногда Редко Никогда

1. **Как часто Вы чувствуете себя уставшим и раздражительным в течение дня?**

Всегда Часто Иногда Редко Никогда

1. **Как часто Вы испытываете трудности пробуждения по утрам?**

Всегда Часто Иногда Редко Никогда

1. **Как часто Вы снова засыпаете после того как проснетесь утром?**

Всегда Часто Иногда Редко Никогда

1. **Как часто Вам нужна посторонняя помощь чтобы проснуться утром?**

Всегда Часто Иногда Редко Никогда

1. **Как часто вы думаете что Вам не хватает сна?**

Всегда Часто Иногда Редко Никогда

Баллы 4 3 2 1 0

Общий итог
